# Environmental Impact of Food Packaging Materials: A Review of Contemporary Development from Conventional Plastics to Polylactic Acid Based Materials

**DOI:** 10.3390/ma13214994

**Published:** 2020-11-06

**Authors:** Lindani Koketso Ncube, Albert Uchenna Ude, Enoch Nifise Ogunmuyiwa, Rozli Zulkifli, Isaac Nongwe Beas

**Affiliations:** 1 Department of Mechanical, Energy and Industrial Engineering, Faculty of Engineering and Technology (FET), Botswana International University of Science and Technology (BIUST), Private Mail Bag 16, Palapye, Botswana; nl19100062@studentmail.biust.ac.bw; 2 Department of Chemical, Materials &Metallurgical Engineering Academic, FET, BIUST, Private Mail Bag 16, Palapye, Botswana; ogunmuyiwae@biust.ac.bw; 3 Department of Mechanical and Materials Engineering, Faculty of Engineering and Built Environment, The National University of Malaysia, UKM, Bangi 43600, Malaysia; rozlizulkifli@ukm.edu.my; 4 Botswana Institute for Technology Research and Innovation (BITRI), Private Bag 0082, Gaborone, Botswana; ibeas@bitri.co.bw

**Keywords:** food packaging, bioplastics, polylactic acid, biodegradable, composites

## Abstract

Plastics have remained the material of choice, and after serving their intended purpose, a large proportion ends up in the environment where they persist for centuries. The packaging industry is the largest and growing consumer of synthetic plastics derived from fossil fuels. Food packaging plastics account for the bulk of plastic waste that are polluting the environment. Additionally, given the fact that petroleum reserves are finite and facing depletion, there is a need for the development of alternative materials that can serve the same purpose as conventional plastics. This paper reviews the function of packaging materials and highlights the future potential of the adoption of green materials. Biopolymers have emerged as promising green materials although they still have very low market uptake. Polylactic acid (PLA) has emerged as the most favoured bioplastic. However, it is limited by its high cost and some performance drawbacks. Blending with agricultural waste and natural fillers can result in green composites at low cost, low greenhouse gas emissions, and with improved performance for food packaging applications. The continent of Africa is proposed as a rich source of fibres and fillers that can be sustainably exploited to fabricate green composites in a bid to achieve a circular economy.

## 1. Introduction

Usage of non-biodegradable materials for the various packaging applications has raised environmental pollution concerns [1,2]. Food packaging accounts for the biggest growing sector within the synthetic plastic packaging market domain [3,4,5,6,7]. Large amounts of different materials, like paper, glass and plastics, are used globally to manufacture packaging materials and more than two thirds are used in the food sector alone. This amount is growing unceasingly as a result of changes occurring in habits of food preparation and consumption, as well as the positive development of various areas and markets in the world [8]. The packaging industry consumes the highest volumes of plastics produced globally and is the main source supplying waste plastics into the environment at an alarming rate [9]. This can be attributed to single-use plastics and the increase of on-the-go snacks and ready-made meals that imply the once-off use of durable plastic packaging material. As a result, there is increasing need for eco-friendly sustainable packaging materials with the desired physical, mechanical and barrier properties for food packaging.

Several studies have reported on PLA as a potential biopolymer to replace conventional plastics in various application including packaging. This review focuses on the function of PLA-based packaging as well as the impact of food packaging waste in the foreseeable future as a result of forecasted global growth. The use of composites from natural fibres and fillers with biopolymers is a fast-growing area. This review reports for the first time the myriad of possible PLA-based composites that could be fabricated from exploiting the variety of plant waste from Southern Africa. It also summarises some recent studies that further argue for the biodegradability of virgin PLA in the marine environment.

## 2. Food Packaging

Petrochemical plastics have found the most usage in food packaging industries because they are cheap, have good tensile properties, and represent an effective barrier against oxygen, carbon dioxide and water vapour. A wide variety of these plastics have been used in packaging in both flexible and rigid form. These plastics can be classified as thermoplastic or thermosets. Thermoplastics can be processed and reprocessed using heat. The ability to reprocess this group of plastics makes them recyclable, as they can be easily moulded to different shapes, and as such, are more ideal for food packaging. Thermoplastics most widely used in food packaging materials are low-density polyethylene (LDPE), polypropylene (PP), polyvinyl chloride (PVC), polyethylene terephthalate (PET), high-density polyethylene (HDPE), polystyrene (PS), and expanded polystyrene [10,11]. On the other hand, thermosets cannot be reprocessed by heat once they are formed. Thus, they are not recyclable and are not often used in food packaging. [12,13].

It has been estimated that, by 2050, a 50% increase in global food supplies will be required due to the increase in global population growth [14]. As demand for food rises, so does the demand for food packaging materials. Packaging materials need to be tailored to be able to maintain the quality of food as well as other rising demands from the consumers, producers, as well as legislative forces. Such demands have grown very dynamic, with calls not only for the best quality of food, but also for that food to be delivered using a sustainable packaging that imposes less impact on the health of consumers as well as the environment.

### 2.1. Packaging Waste in Food Industry

**Figure 1:**
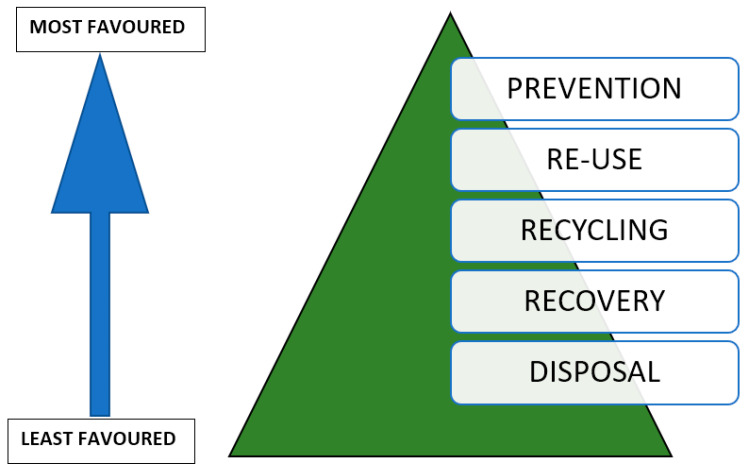
EU Waste Management Hierarchy. Adapted from [18].

In the packaging industry, food packaging accounts for 50% of the plastics derived from fossil fuels [11]. When food is thrown away, so does the packaging material where it was contained. These fossil fuel plastics are persistent in the environment and take many years to degrade. As they do, they break into microplastics, which can easily enter the food chain when consumed by, for example, fish, leading to bio accumulation. Growing environmental concerns have placed packaging under scrutiny as it is a constant source of high amounts of plastic waste and this has brought about the need to do extensive research into renewable alternatives [15,16,17]. A five-step waste management hierarchy was defined by the European Union (EU) Waste Framework Directive 2008/98/EC as shown in Figure 1. The hierarchy ranks the treatments of waste based on the ability to conserve resources, with the prevention or minimisation of waste being the most preferred route to follow into the future, and the disposal of waste being the worst-case scenario to be avoided at all costs.

Packaging materials in use today are chiefly fossil fuel-based plastics and their annual production continues to rise [19]. Therefore, it is imperative to bear in mind that the best solution to lower plastic waste in the environment is better waste management, particularly in developing countries [20]. The adoption of green packaging is vital and three types of green packaging have been identified namely [21];

1. Reusable packaging, e.g., glass which can be reused after cleaning.
2. Recyclable packaging, e.g., paper which can be reprocessed and reused.
3. Biodegradable packaging, e.g., cotton sacks which can break down into the environment without causing damage.

### 2.2. Biodegradable Food Packaging

The food packaging industry is now in pursuit of biodegradable packaging that is lightweight for reducing materials use, waste and as well as transportation costs. Plastics from biopolymers are promising to fulfil this requirement. Biodegradation is a process of defragmentation, initiated by heat, moisture, and/or microbial enzymes, which transforms longer molecular substances into smaller compounds [22]. It can also be simply defined as a process by which substances are broken down by living organisms [2,23,24]. A number of studies have been conducted on the possible utilization of bio-based materials in the pursuit of developing sustainable packaging materials. Although some improvements have been achieved, a balance among environmental concerns, economic considerations and product packaging performance is also still lacking [1,25,26]. The exploitation of natural biodegradable polymers and their blends for the green synthesis of polymer-based packaging material can provide a sustainable solution due to the numerous advantages of these polymers, including low cost, accessibility, biodegradability, and flexible processability, to improve and develop new sets of polymeric materials with desired properties [3,27,28,29,30].

#### Sustainable Food Packaging

The objective of sustainable food packaging is to integrate functional and/or innovative materials into packaging that can improve economic and environmental health [1,31,32]. Recyclable materials can also be employed together with the use of lifecycle assessments and lifecycle inventories to minimize the ecological footprint and environmental impact of the resulting packaging. Sustainable food packaging serves to address the plastic waste accumulation in the environment, reduce consumption and/or dependency on finite fossil fuel reserves, and minimise food waste, thus saving food resources [14]. However, sustainable packaging is still a multifaceted concept that necessitates investigations and documentation to appraise the package design, materials used, processing, and life cycles.

In response to the need of sustainable packaging, the Granting society with low environmental innovative packaging (GLOPACK), a European 2020 innovation action is set on developing new generation bio-based and fully degradable composite food-trays made of biopolymers and fibres from agro-wastes. The bio-wastes used are residues from the processed fruits and vegetables industry for producing biopolyesters for use as the matrix. The lignocellulosic fillers are solid residues from the wheat industry which are blended to the matrix and processed using hot melt extrusion to produce the composites [33].

Food packaging is generally viewed as an unnecessary cost [34]. It is considered as an additional economic and environmental cost instead of rightfully being seen as an added value for the reduction of food loss by improving shelf life. It is reported that at least 20% food waste is a result of errors or misinterpretation of food date labels, and this shows the importance of the packaging in achieving several functions [14]. Packaging material and the product to be packaged should be considered as one system from the design phase of the material to match the properties of the food. Sustainable of green packaging development using edible or biodegradable materials and their composites, plant extracts, and nanomaterials can help to bring down the environmental impacts of food packaging [35].

## 3. Biopolymers

Biopolymers are those polymers that are produced by living organisms or derived from biomass and after serving their purpose, they degrade within a reasonable time period without causing environmental waste problems [36,37]. They are made up of long chain compounds, composed of of long chain molecule subunits, and they make up much of living bodies and the majority of the biosphere. These biopolymers can either be thermosetting or thermoplastic polymers [38]. Biobased polymers can be [2,20,26,39,40,41,42,43]:

1. directly extracted from biomass (for example polysaccharides such as starch or cellulose),
2. produced by chemical synthesis using renewable biobased monomers (for example polylactic acid),
3. produced by microorganisms or genetically modified bacteria (for example polyhydroxyalkonoates),
4. and/or by chemical synthesis using both bio-derived monomers and petroleum-based monomers (for example poly(butylene succinate)).

Biopolymers mimic the properties of conventional polymers and can be processed using a several methods including solution casting, melt-mixing, electrospinning, thermo pressing and extrusion [44]. Polymers generally make use of additives during processing. Therefore, biodegradable packaging polymers should be used with biodegradable additives so as not to compromise their biodegradability [20]. Bio-based polymers have not only found many applications replacing existing polymers, but can also provide new combinations of properties [45]. Biobased materials can be particularly valuable in the three main areas of food-related applications, namely in food packaging, food coating, and edible films for food and encapsulation [46]. These biopolymers used to replace petrochemical plastics are also referred to as bioplastics.

Adoption of biopolymers as food packaging materials should no longer be viewed as an option but as a necessity [47]. The widespread use of biodegradable materials for packaging can result is reduced plastic waste, lower greenhouse gas emissions and guarantee the sustainable exploitation of environmental resource [44,48,49,50]. In the medical field, where function is more important than cost, biopolymers have found important applications and have established themselves well. Biopolymers are thought not likely to replace all fossil fuel based polymers for packaging applications where cost has been viewed as more important than environmental issues [14,22,36]. This is a result of some drawbacks in terms of thermal resistance, as well as barrier and mechanical properties. As such, more research is necessary to meet the packaging requirements at acceptable cost.

### 3.1. Classification of Bioplastics

**Figure 2:**
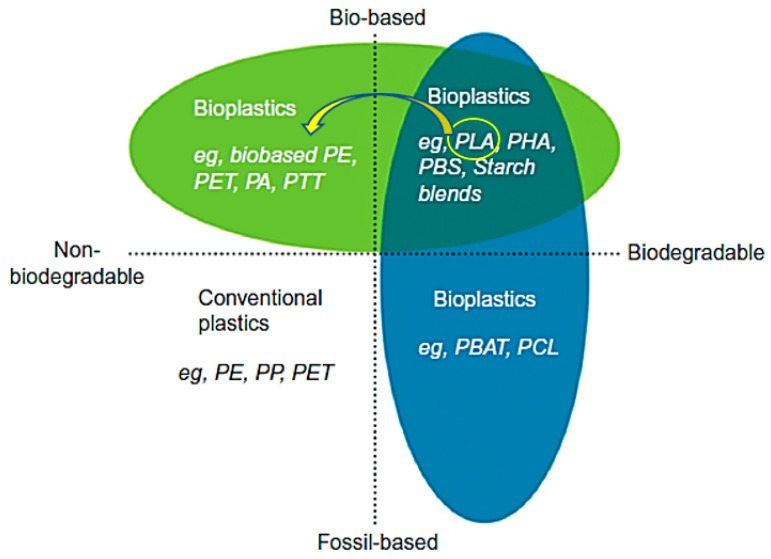
Bioplastics material coordinate system. Adapted from [51].

The term bioplastics can be confusing at times, it could mean plastics are derived from bio sources or that plastics are biodegradable. Some plastics from bio sources are non-biodegradable and there are those from fossil fuels that are biodegradable. Therefore, in this review, the term bioplastics will be used to refer to biodegradable plastics derived from biomass. Bioplastics can be biobased and/or biodegradable. Thus, not all biobased materials are biodegradable and also not all biodegradable materials are biobased. A biodegradable material is one that can be broken down by microbes and used as a food source. The American Society of Testing Materials (ASTM) standards D-5488-94d defines biodegradable as the ability to decompose into carbon dioxide, methane, water, inorganic compounds, and biomass [44]. Flexible packaging and rigid packaging are mostly made from biodegradable bioplastics and from non-biodegradable bioplastics respectively [40]. The commonly used bioplastics are depicted in Figure 2.

PLA is marked with an arrow representing a shift from the status of biodegradable biobased polymer to nonbiodegradable biobased polymer. In literature, PLA is widely reported as a biodegradable polyester that can serve as an alternative to petrochemical plastics [52,53]. However, studies are continually highlighting the slow rate of degradation which can take up to 3–5 years depending on properties such as crystallinity, molecular weight, water absorption, and stereoisomeric content [53]. In addition, the biodiversity of the degrading microorganisms is different under different environmental conditions and as such degradation rates would be different [54]. PLA has been extensively reported to degrade at a reasonable rate in soil and more efficiently in compost environments [55,56]. However, in the marine environment PLA has performed poorly and even comparable to fossil-based plastics. One study showed that the surface degradation rate for HDPE was similar to PLA in the marine environment although on land PLA degraded 20 times faster [57]. In packaging application, PLA has the advantage of being compostable together with organic waste, even though it would degrade at a slower rate [58]. This means that food contaminated packaging can be sent for composting with no need for cleaning. The challenge comes when the packaging waste enters water bodies into the oceans where there are low temperatures and high pressures presenting conditions that are not conducive for PLA degrading microorganisms [54]. Packaging waste is one of the biggest polluters of water bodies, and as such, there is a need to curb the mismanagement of bioplastic products marketed as biodegradable as they may have similar effects as fossil-based counterparts on marine organisms and biological accumulation [59].

It is paramount to note that non-biodegradable bioplastics from bio sources still have advantages over biodegradable fossil fuel plastics. They have better recyclability and low levels of carbon dioxide production as well as compatibility with existing plastic processing systems [43]. Examples are biobased PE, PP, and PET that have same physical and functional properties as their petroleum counterparts. Moving forward, new varieties of plastics will be made from renewable biomass as a result of the depletion of petroleum reserves [46,60].

### 3.2. Bioplastic Market

**Figure 3:**
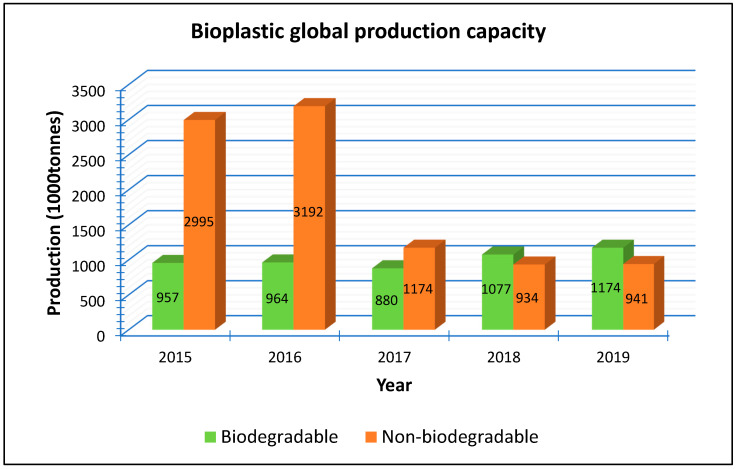
Share of biodegradable and non-biodegradable bioplastics production capacities (2015 to 2019). Data from [64].

The bioplastics industry is reported to be a young, innovative sector possessing a great economic and ecological potential for a low-carbon, circular bioeconomy that utilises resources more efficiently [16,61]. The market for biopolymers is expected to show some growth and new demands are envisioned from applications which have clear benefit to the consumer and the environment [45]. Biopolymer global production has grown from just under 300,000 tonnes in 2009 to 2.11 million tonnes in 2019 and is predicted to grow to 2.42 million tonnes by the year 2024 [62,63]. Bioplastics produced in 2015 globally had the shares of biobased non-degradable and biodegradable plastics at about 64% and 36%, respectively [40,44]. There is envisioned growth for the share of biodegradable polymers to meet environmental demands of reduced pollution and in 2019, the share for biodegradable plastics grew to about 56%. Figure 3 illustrates the drop in non-biodegradable share and rise of biodegradable bioplastic share.

**Figure 4:**
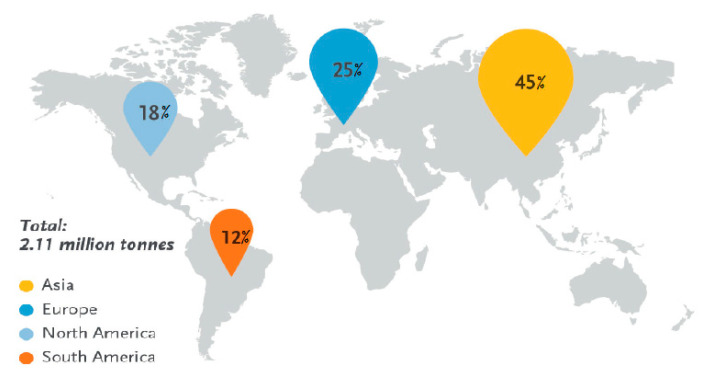
Global production capacities of bioplastics in 2019. Adapted from [65].

Figure 4 shows the global production capacities of bioplastics in 2019. Asia was the major producer accounting for 45% of global production. Land used to grow renewable stocks for bioplastic production stood at 0.79 million hectares which is 0.02% of the global agricultural area and this will remain the same despite the predicted growth of bioplastic production. As such, this will have no impact on the 97% of global agricultural area which is being used for pasture, feed, and food [65]. Africa is yet to make its mark as it is still lagging behind in terms of production of bioplastics, despite the availability of raw material resources.

### 3.3. Use of Biopolymers in Developed Countries

In developed countries, biopolymer materials have managed to replace conventional counterparts as food packaging material for mostly organic, natural and functional foods [26]. Biopolymers have greatly influenced the packaging sector as a result of increasing importance of environmental responsibility to both consumers and industry. As a result, packaging is the biggest consumer of biodegradable bioplastics, which in 2019 accounted for 1.14 million tonnes which is more that 53% of the total bioplastics market [63]. These biopolymers limit carbon dioxide emissions during creation and after disposal they degrade to organic matter. Biodegradable polymers can, without any threat to the environment, retard deterioration, extend shelf life, and maintain quality and safety of packaged food [39]. Despite their high cost of production, bio-based products are undoubtedly the fundamental concept of a circular economy [66]. As a plus, the use of biopolymers to remedy the environmental pollution from fossil based non-biodegradable polymers can result in an efficient waste management system envisioned globally.

Environmental benefits that come with the use of biopolymers alone have been reported not to be enough to create a big market [22]. Biodegradable polymers have replaced only about 1% of global plastics [10,67]. Therefore there is still great need for basic and applied research to improve their performance, reduce cost and improve their ease of production [44]. The adoption of biodegradable polymers for packaging can provide a solution to waste-disposal problems associated with the traditional petroleum-derived packing materials as well as address the fact that crude oil and natural gas resources are limited [7,25,68,69,70,71]. It is expected that, by 2022, the use of biopolymers in food packaging should have grown by 25% [11].

Several biopolymers that can be used for packaging purposes include starch, cellulose, chitosan, polylactic acid (PLA), polycaprolactone (PCL) and polyhydroxybutyrate (PHB) among many others. Products like bottles, jars, cans, buckets, food containers, disposable cups, packaging films, and refuse bags can be manufactured using biodegradable polymers [39]. Among bioplastics, PLA polymer has been shown to possess the properties that enable it to compete with petroleum-based plastics in various application areas [30,41,72,73,74]. Thus, this review is biased to PLA and its biocomposites.

## 4. Polylactic Acid

**Figure 5:**
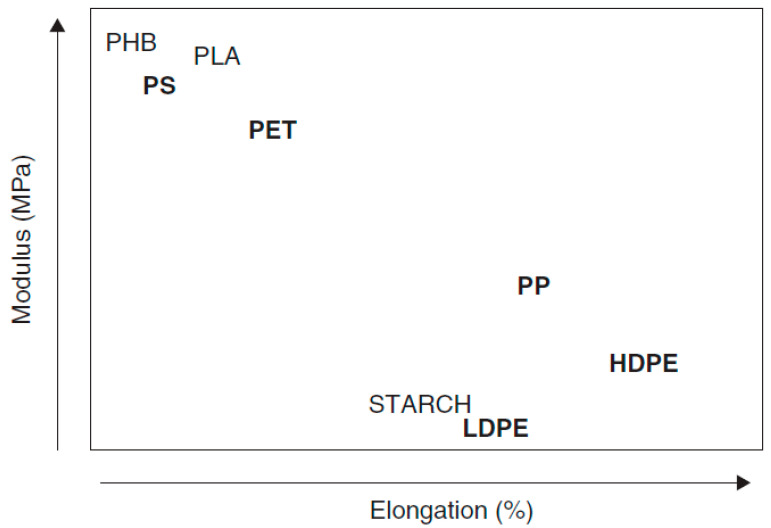
Mechanical properties of biopolymers and fossil-based polymers [28].

Polylactic acid (PLA) is the most promising material with a lot of research potential. It presents packaging applications for a wider array of products including films, forms, food containers and coatings among many others [2,36,50,75]. PLA is environmentally friendly biodegradable thermoplastic polyester. When disposed into the environment, pure PLA can slowly degrade over a period of months to two years whilst petroleum based plastics take more that 500–1000 years [76,77]. Also, by composting with other biomass such as compost soil, PLA can completely degrade within 3–4 weeks [49]. PLA possesses a wide range of desirable properties including biocompatibility, favourable mechanical properties and it can be moulded into various shapes making its performance comparable to petroleum-based plastics [24,49,74,78,79,80,81]. For example, a PLA bottle is comparable to a PET bottle as the tensile strength and elastic modulus of the two polymers are comparable. Whilst the production of the PET bottle, when compared to the manufacture of the same bottle using PLA consumes 36% less energy and produces 44% less carbon dioxide [43]. Thus, the use of PLA brings in environmental benefits with similar performance as traditional PET. A comparison of mechanical properties (modulus and elongation at break) of some plastics from biobased polymers with those of fossil-based plastic polymers is shown in Figure 5. In the figure, fossil-based polymers are shown in bold.

### 4.1. Production of PLA

This thermoplastic polyester can be derived from fermentation or by chemical synthesis [37,43,70];

1. Fermentation of carbohydrates such as starch. The bulk of PLA produced is from the crops corn and cassava which are renewable resources.
2. Chemical synthesis uses ring opening polymerisation reactions to obtain high molecular weight polymers.

**Figure 6:**
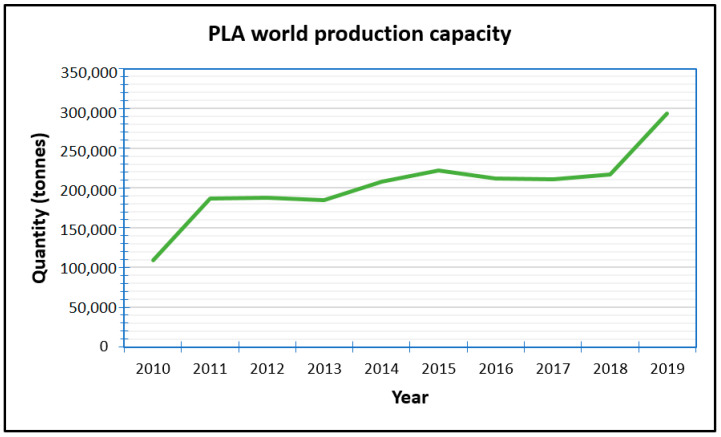
World production capacity for PLA (2010 to 2019). Data from [64].

Lactic acid (LA) monomer, used for the production of PLA, was first produced commercially in Germany under pharmacy Boehringer Ingelheim in 1895 [82]. PLA has three structures namely the poly(L-lactide) (PLLA), poly(D-lactide) (PDLA), and poly(D,L-lactide) (PDLLA). This is because it has two stereoisomeric forms of lactic acid which are laevo- or L-lactic acids and dextro- or D-lactic acids. The ratio of combination of these enantiomers and their sequences have a bearing on crystallinity, thermal and mechanical properties of the resulting PLA [70,83]. Increasing the D-lactide concentration produces high cost PLA polymers with a more crystalline structure and films with better thermal stability, mechanical strength, and barrier properties. Poly(D,L-lactide) with 88–90% L-lactide has been widely used for packaging purposes as it is easy to process by thermoforming and displays properties similar to polystyrene [43,70]. PLA can be processed into films, sheets or moulded products by employing methods such as extrusion, thermoforming, injection- or blow- moulding, and film blowing or stretching. Biodegradable and non-biodegradable plasticizers have been used with PLA to improve processability, although only nontoxic ones are suitable for food contact applications [84]. Packaging products from PLA include shopping bags, cups, trays, wrapping, films, containers, foams, and bottles. Over the years the production of PLA has been steadily on the rise as can be seen from Figure 6 and estimates indicate that it would rise to just over 300,000 tonnes by 2024.

### 4.2. Lifecycle of PLA

**Figure 7:**
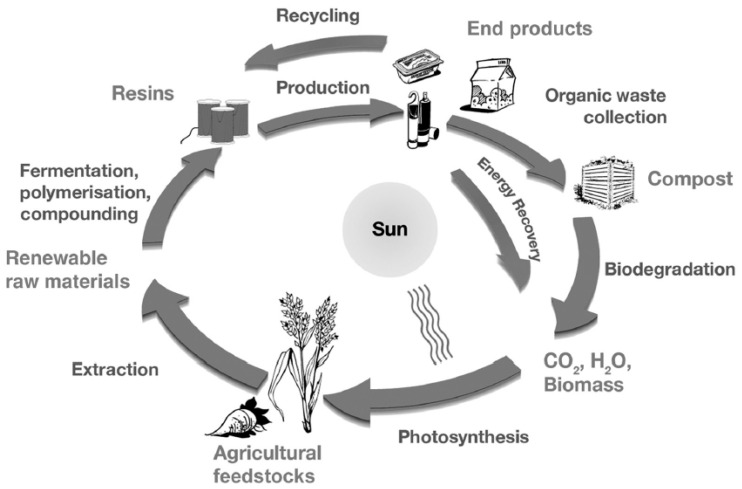
Lifecycle of PLA bioplastic [83].

The bioplastic PLA generally follows the same route as all other biodegradable bioplastics. PLA is manufactured from renewable resources and converted into consumer products. In food packaging, these products are mostly single-use primary packaging plastics for ready meals. They are characterised with short service lifetime and these products include cups, bottles, clam shells and wrappers. Thus, their degradability is an important factor as they easily find their way into the environment as a result of the human throwaway culture. Replacing non-biodegradable plastics with biodegradable PLA will go a long way in environmental protection. Figure 7 depicts the lifecycle of the polymer.

Generally, waste management calls for products that have served their purpose to be reused, recycled, incinerated, and disposed into landfills. Recycling of PLA is environmentally beneficial as shown by investigations in almost all environmental impact categories [85]. Thus, from an environmental point of view, composting is the most favourable end of life option for PLA as it is considered as an organic or biological recycling option for biodegradable plastics [20]. Composting is the controlled aerobic or biological degradation of organic materials to generate a carbon and nutrient rich compost that acts as a natural fertiliser than can be used to grow crops and thus reducing the demand for chemical fertilisers [13,83]. PLA degradation happens mainly through the scission of the backbone ester bonds and in humans and animals PLA undergoes hydrolysis to form soluble oligomers that can be metabolised by cells [37,43,86]. The use of PLA results in an intrinsic zero material carbon footprint value [87].

### 4.3. PLA Properties of Interest to Food Industry

Food packaging material is in direct contact with food and more that 6500 chemicals are used in manufacture of food contact materials [10]. Depending on the material used, chemicals can leach into food through a process referred to as migration and these chemicals may pose risk to consumers [10,88,89]. The Food and Drug Administration (FDA) approved the LA monomer as a safe food ingredient, and this made PLA a green polymer of interest that possessed low toxicity as a bonus [28,29,74,84,90]. PLA is a safe material for food packaging applications as the migration of LA from PLA packaging containers to food is insignificant. PLA possesses a number of desirable properties that make them ideal for application in food contact packaging materials. These properties are listed below [28,37,43,46,74,83];

- Excellent transparency, which is one property of interest in food packaging.
- Permeability to carbon dioxide compared to that of oxygen (perm selectivity) is higher than that of most conventional fossil fuel-based plastics. This is particularly important in food packaging applications where high barrier to oxygen is required.
- 20 times better oxygen barrier properties than polystyrene (PS).
- Better mechanical performance that PS.
- Relatively good water resistance.
- Good chemical resistance to fats and oils.
- Better thermal processability compared to other bioplastics, as seen from its relatively high glass transition temperature and low melting temperature.
- Films seal well at temperatures lower than melting temperature.
- PLA films also show better ultraviolet light barrier properties than low density polyethylene (LDPE).
- In addition to being biodegradable, PLA can also be recycled or incinerated. Using steam or boiling water, PLA can be hydrolysed to lactic acid, leading to molecular recycling, allowing recycling of packaging materials.

The main application of PLA has been mostly limited to medical application as a result of the high cost of the polymer, low availability, and limited molecular weight. However, new techniques for the economical production of PLA are contributing to the growth of utilisation in packaging and textile sectors [2,45,81]. Also, as a result of its economic and commercial viability during processing, the biopolymer PLA has attracted a lot of attention as a substitute to conventional polymers to serve as a sustainable green material that can perform better [40,46]. However, in molten state, PLA tends to thermally degrade posing a processing challenge [30,46,91]. Also, the mechanical and barrier properties do not match fossil-based polymer counterparts at times. It can have excessive brittleness and unsatisfactory barrier to oxygen and to water compared to benchmark polymer PET [46]. PLA can present challenges in rigid thermoformed packaging as a result of its low deformation at break, high modulus and hydrophilic properties [43]. All these challenges can be overcome by employing various methods, including blending with other polymers, making composites, as well as coating and polymer modification [24,36,74,75].

### 4.4. Biodegradable Composites Based on PLA

Composites are made from combining different materials in effort to improve physical, chemical, mechanical and processability properties [30,83,92]. Cost reduction can also be achieved using this blending of materials. The common method of composite manufacture using PLA is melt blending with the desired fillers and the final products are produced using extrusion, blow film moulding, and compression- and injection-moulding [11]. For modification of functionalities to improve its properties, it can be incorporated with other materials including starch, other bioplastics, nano clays, carbon nanotubes, and cellulose [43,83].

A growing area of polymeric composites is in the development of ecologically viable materials with less environmental impact using raw materials from natural sources [30,93,94,95]. The composite manufacturing industry is now more inclined to the adoption of natural fibre reinforcements to replace synthetic fibres, and fibres such as flax, hemp, jute, sisal, kenaf, pineapple, abaca, coir, and banana have been used. As a result of their low cost, biodegradability, large availability, and valuable mechanical and physical attributes, a wide variety of lignocellulosic fibres and natural fillers from agricultural and industrial crops, such as corn, wheat, and bagasse, have been employed in the production of composites for areas including packaging, automotive and building industry [30,96,97,98]. Plastics used for composite production account for only 4–5% of the total plastics produced [61]. As such, opportunities exist to complement the draw backs of biodegradable polymers as well as reduce the cost of the composite materials for the packaging industry.

Natural fibres are generally non-toxic and can be safely utilised without posing any health hazards or environmental damage. Plant-based fibrous materials used with biopolymers/biomass-derived polymers can yield environmentally friendly and biodegradable green composites with sufficient flexibility and mechanical strength comparable to commercially available petroleum-based polymers [71,99,100,101]. As a result of its inherent drawbacks, PLA can be used with numerous organic fibres and fillers to improve some of its properties such as mechanical strength and reduced gas or moisture permeability. These fillers can include cellulose in different forms and of different origins, as well as untreated or modified wood flour, kraft lignin, or wastes deriving from olive pit, or waste from wine production as well as waste deriving from coffee production among many others [98,102,103,104,105]. Also, the complete biodegradation of PLA in soil occurs at a relatively slow rate and the addition of natural biodegradable fillers can catalyse a speedy biodegradation of the resultant PLA-based composites [106]. For example, blending with kraft lignin accelerates biodegradation of PLA in garden soil [77].

The following Table 1 shows some research on composites from PLA with natural fillers to improve its properties as well as reduce the cost of the final composite. With a high amount of agricultural and food waste, novel composites can be fabricated using this waste with biopolymers resulting in a reduced environmental burden.

### 4.5. Properties Required of Materials in Food Packaging

The properties that are of importance to food packaging application material are thermal, mechanical, chemical reactivity, optical, gas and moisture barrier properties [11,22,109]. The packaging material must be able to maintain optimum function without compromise on the durability and strength properties during storage of food until disposal [26,76,110]. Matching the durability of the biobased primary packaging with product shelf life has been one of the biggest challenges in the food packaging industry. Green composites based on PLA and natural fillers and fibres from agro waste may be investigated to serve as sustainable packaging. Table 2 shows some properties of PLA compared to other common polymers used in food packaging.

#### 4.5.1. Thermal Properties

Good thermal properties protect products against thermal damage during storage and/or resist deformation and degradation of packaging materials as they store products with elevated temperatures. Synthetic fibres and fillers generally have better thermal stability and thus their composites have better heat resistance. Natural fibres degrade at elevated temperatures affecting their range of application, choice of matrix and processing routes [38]. Natural fibres are also more flammable than their synthetic counterparts and as such make their composites more flammable [49]. Introduction of flame retardants to counter this problem can lead to reduced durability and as well as to toxic products after thermal degradation of the composite. The constitution and sequence of PLA monomers can give PLA with higher tolerance to temperature, allowing the packaging material to satisfactorily accommodate hot meals.

#### 4.5.2. Mechanical Properties

Required mechanical properties ensure the packaged products are protected from external forces and/or ensure the packaging material has adequate strength to contain the packaged goods [109]. Bioplastics have different strength properties and depending on end usage they may be found appropriate or lacking. Blending with other materials can help to improve the resultant composite mechanical properties. To achieve a cost-effective biodegradable composite, bioplastics can be blended with other biodegradable materials such as natural fibres. Natural fibres suffer from variability in properties as they are from natural sources. Factors that result in variation include crop variety, seed density, soil quality, fertilisation, field location, fibre location on the plant, climate, and harvest timing [38]. As such, mechanical properties may be difficult to accurately predict. However, the benefit of improved mechanical properties as well as biodegradability makes lignocellulose natural fillers and fibres the best low-cost material for blending [30]. Also, the interfacial adhesion between the fibre and matrix affects the final mechanical properties, with good adhesion resulting in improved strength [114].

#### 4.5.3. Chemical Reactivity

Packaging products should have inertness as it can contain even strong acid characteristic products that could compromise other properties such as mechanical properties thus compromising function [70]. Also, migration of organic and/or inorganic chemicals from packaging materials to food is another important chemical property. The production of packaging materials can result in several chemicals being present in packaging materials as a result of chemical reaction by-products, degradation of additives, as well as the presence of impurities, plasticisers, and antioxidants [10,109]. Chemical migration mostly depends on the type of material used in contact with food, properties of the migrant and as well as the food [89]. Food contact materials have been found to be a potent source of chronic exposure to chemicals [88]. Migration is even possible from inert materials like glass, ceramics, or stainless steel where chemicals on the surface in contact with food interact with it [10]. However, use of PLA based materials can lead to the reduction of exposure to these problematic migrating chemicals as it releases harmless negligible amounts of LA, a substance that is also found in human bodies. Blends with other non-toxic naturally occurring materials provide safe packaging composites.

#### 4.5.4. Optical Properties

Transparency of packaging can allow consumers to see the product contained in the package. The consumer can then judge the product quality using its appearance. This is particularly important for foods like fresh meat, confectionery, fruits, and vegetables. However, deteriorative reactions on food and packaging polymer could be catalysed by light [109]. Plastic polymers maybe opaque, hazy, or transparent. PLA has high transparency and surface gloss as such it is a clear material that can easily show the packaged product [83]. Blending with cellulose fibres has been reported not to have negative effects on the colour of the composites, thus preserving the desirable appearance of PLA [30].

#### 4.5.5. Gas Barrier Properties

In order to retain quality of food during storage, certain gas compositions must be maintained inside the package. These gas mixtures comprise of oxygen, nitrogen, and carbon dioxide. Oxygen content is a critical factor as it contributes to oxidation leading to rapid spoiling of food [101]. Biopolymers are generally hydrophilic and as such an increase in humidity results in an increase in gas permeability. An increase in crystallinity of the polymer results in improved barrier properties [22]. Thus, the utilisation of PLA with higher crystallinity would yield improved gas barrier properties to control the penetration of air though the packaging material thereby maintain the quality of food thus extending the shelf life to. The plasticisation of PLA can also significantly reduce the oxygen transmission rate, and as such reduce the oxidation of packaged food [84]. This will avoid food losses which is another objective of sustainable packaging.

#### 4.5.6. Moisture Barrier Properties

Moisture barrier is the ability to prevent undesired vapour to pass through the packaging material. This property is affected by permeability, diffusivity, solubility across the barrier, and affinity towards moisture of the packaging material [22]. Moisture barrier properties of biobased packaging materials are generally inferior to those of conventional materials [110,115]. The moisture vapour transmission rate of PLA is up to five times greater that its fossil-based counterparts [115]. Increased moisture barrier increases shelf life of food in food packaging industry. It goes without saying that moist foods would have reduced storage periods as the stability of the packaging material will be compromised. PLA has carbonyl groups which are responsible for the poor moisture resistance [106]. The introduction of moisture promotes the onset of biodegradation of PLA polymer. The blending of PLA with natural fibres and fillers further promotes the degradability of the resultant composite [30]. This makes the composites ideal for single-use packaging by eliminating the waste disposal challenges [116].

#### 4.5.7. Water Absorption

Green composites in a moist environment can promote the growth of bacteria and fungus that results in rotting. This problem is a result of the inherent nature of biopolymers which absorb more water compared to fossil-based counterparts [38]. This problem is compounded by the use of natural fibre fillers which by nature are hydrophilic particularly cellulosic fibres [2]. Hydroxyl groups present in these fillers initiate and promote the degradation of PLA [117]. The higher the water content absorbed, the more the promotion of food oxidation leading to food spoiling [108]. However, as these materials are meant for single use within a short time, should they be inadequately disposed of after use into the environment, the exposure to water would mean accelerated biodegradation, which is what is needed.

#### 4.5.8. Durability

Biodegradable materials are generally characterised by limited durability whilst their petroleum-based counterparts are more durable. This poses a limitation in application potential, but it is a plus for applications that need to eliminate environmental pollution when the materials are disposed into the environment post use. Biodegradable composites are therefore more suitable for short lifespan products [38]. These materials would be ideal for single-use packaging applications and their demand is likely to grow [30]. Exposure to environmental conditions results in degradation of the green composites. For example, exposure to ultraviolet light subjects the composites to photoirradiation which disrupts the chemical bonds of the organic polymers resulting in the compromise of physical and mechanical properties [49]. It is reported that more studies are still required to assess how the process of biodegradation affects properties of PLA-based composites for food packaging [116].

### 4.6. Biodegrable Packaging Influence on Food Products

Biodegradable packaging materials can successfully serve as alternative for conventional fossil-based packaging materials [118]. Their use has grown to address advanced functionality packaging applications like active packaging and modified atmosphere packaging applications. Some examples of note are briefly discussed on the use of virgin PLA and its composites and their influence on packaged food.

Virgin PLA can satisfactorily serve as packaging material of choice in applications that do not need modified atmospheres for example for fruits and vegetables under ambient conditions [119]. The natural properties of PLA are sufficient to simply contain the foods.

A compostable PLA and cellulose based packaging for fresh-cut cherry tomatoes was developed by [120] and compared to PET tray and PP film. The compostable packaging prolonged the shelf-life of fresh-cut cherry tomatoes. The packaging also displayed better properties that were enhanced by ultraviolet (UV) sterilisation treatment. For example, it displayed high water vapour transmission rate resulting in an anti-fog effect which abated microbial growth for extended periods of time than the fossil-based plastic counterpart.

Active packaging films have been developed by [121] using PLA and bleached bagasse carboxymethyl cellulose for mango storage life extension. Mango fruits have high rates of perspiration which result in the deterioration of their freshness. The resultant packaging film composite had good film flexibility and managed to increase the shelf life for mangoes for 42 days as a result of improved moisture control thereby reducing the respiration rates of the fruits.

PLA trays wrapped with PLA films were also investigated by [90] for use as a packaging solution for refrigerated fresh red meat. These were compared to conventional packaging from amorphous polyethylene terephthalate/polyethylene trays wrapped with polyvinyl chloride plastic. The PLA packaging system managed to maintain the fresh red colour longer that the conventional packaging material effectively extending the shelf life by two more days. This was a result of the microbiological sensory and chemical attributes of PLA.

Processed meat packaging from PLA nanocomposite films embedded with unmodified montmorillonite were developed by [122]. The developed films were used to package sliced salami, a fatty food. As a result of enhanced water barrier properties, the nanocomposites were able to retard lipid oxidation of the packaged processed meat, effectively extending the shelf life.

Active packaging for bread was developed by [123] using PLA and Poly(butylene-succinate-co-adipate) films containing thymol. The developed material was evaluated for bread fungal protection compared to petroleum based biaxially oriented polypropylene (BOPP), a common bread packaging material. Mold growth in the biodegradable composite was witnessed after nine days in storage, whereas in BOPP, it was after three days, showing an improvement in shelf life. Yeast and mold counts were correlated to the amount of carbon dioxide which was higher in BOPP.

### 4.7. Biodegradation Routes

Several end of life options are available for biodegradable composites for food packaging. The routes of note are biodegradation in soil, compost, and aquatic environments since most of the packaging developed would be single use and easily find its way into the environment or into compost facilities. The growing commercialisation of PLA for short-shelf life products has warranted an increase in studies of its degradation [81].

#### 4.7.1. Soil

Packaging waste is a notable land pollutant. Landfilling is a conventional waste management approach where a great amount of waste is disposed in [124]. This soil environment has a vast amount of microorganisms which are responsible for biodegradation of buried waste [54]. Virgin PLA takes relatively longer to degrade in soil compared to its biocomposites. A study by [56] confirmed that PLA and its composites can degrade in soil and the rate of degradation can be regulated by alkali treatment of the cellulose fibre reinforcement and as well as the fibre loading. Soil burial biodegradation of PLA and starch composites were studied by [92] for 14 and 28 days also reveal the presence of biobased fillers improves the biodegradation of PLA.

#### 4.7.2. Compost

Composting involves biodegradation under controlled conditions [115]. It is an important route for the food and catering industry [14]. Most food packaging are contaminated with food which presents challenges to clean off in attempt to recycle the materials and as such composting offers a simpler route that does not require contaminant cleaning [81]. It can be carried out at home or in an industrial set up. Industrial composting involves higher temperatures and the rate biodegradation is higher [54]. The packaging waste is mixed with mature compost at exposed to optimum oxygen and moisture conditions at a temperature of 58 °C [125]. To further accelerate biodegradation rate in compost environments, bioaugmentation can be utilised. A study by [58] enhanced the biodegradation rate of PLA films and its bio nanocomposites in simulated composting through bioaugmentation with Geobacillus microbes.

#### 4.7.3. Aquatic Environment

Fossil-based plastic packaging waste have caused untold ocean pollution [37,47]. It goes without say the importance of investigating the behaviour of biodegradable packaging in marine environment. Not many studies have been done on PLA microbial attack in aquatic systems. The degradation is mostly as a result of hydrolysis and not from microbes [81]. A study of interest was conducted by [59] on the effects of single use cups and plates made from PLA compared to PET in the marine environment. The results demonstrated a possibility that biodegradable products may have similar effects as fossil-based plastic packaging in the aquatic environment, thus warranting more research on the subject.

## 5. Outlook of Biobased Biodegradable Polymers

The rise in global population will demand more food and hence more packaging. Lack of sustainable packaging materials would be detrimental to the environment as there would be increased use of persistent petrochemical based single use plastics. The damage will be more severe especially for developing countries lacking waste management policies and systems. A lack of sustainable packaging will also see the continuation in the rise of food waste that could have otherwise been avoided. In Europe alone, the uptake of sustainable packaging would eventually reduce food waste by 50% in 2050, translating to 100 million tonnes of food saved [14]. Sustainability could be realised from the use of agricultural and agro-food residues to produce naturally biodegradable packaging [14]. Also, upon serving its purpose, the biodegrading recycling method would yield raw material to feed back into the cycle as nutrients for the plants [14]. These products will contribute to the development of a circular bio economy.

For consumers, improving safety and quality of food is an important factor [48]. This requires food packaging with a good barrier against microbes, water vapour, oxygen, light, mechanical contaminants and other external factors that affect the properties of the product [101]. However, most cases of bio-based packaging material demonstrate poor properties that limit their application in food industries [25,41,69,70,126]. Also, cost is another limiting factor hindering the use of biobased packaging materials and it is hoped that, as economies of production improve, costs will be competitive [127,128]. To counter these limitations, polymers can be used as a matrix form composites that can possess desirable properties [49,116,129,130,131]. PLA challenges [30,66,71,98,132] can be addressed with the use of low-cost fillers and fibres from natural sources to produce green composites.

Just over the past four decades, research has also witnessed an intensification in the use of plant fibres as reinforcement for the production of high performance, renewable, and sustainable green composites. These composites are envisioned to bridge the property-performance gap between renewable polymers and petroleum based polymers [49,100]. Southern Africa has some of the richest vegetation on earth and has since been recognised as one of the most fascinating and vital areas of the world from an ecological and evolutionary point of view [133,134]. This variety of African indigenous plants may be sustainably exploited as raw materials for the manufacture as well as reinforcement of biodegradable polymers to provide novel materials for application in different areas as qualified by their resulting attributes. Examples include:

1. In Zimbabwe, a total of 206 wild food plants were documented in one study, and from the findings, only fourteen popular food plants were identified with commercial potential [135].
2. Another study in South Africa on the status of underutilised crops revealed that most are drought tolerant, nutrient dense, and possess the potential for success in addressing food security, climate change adaptation, and improving rural livelihoods. As such, the drive is to increase their production as well as prioritise them for future research, development, and innovation [136].
3. In 2010, a study in Botswana revealed ongoing pilot farmer-based cultivation for selected threatened and indigenous species of medicinal and edible wild fruit plants for lucrative avenues for income generation as the world demand for plants as sources of drugs and novel foods increased [137].

Plant resources provide subsistence and income for millions of people, mostly in developing countries. For each given area setup, the local vegetation provides people with food, fuel, medicine, materials for construction, manufacturing of crafts among several other products [138]. All these indigenous plants, either wild or cultivated, have waste that can be investigated for sustainable utilisation for various applications. This presents opportunities for future research to characterise these materials and find value addition routes for new product development.

The enhancement of mechanical properties of polymers by the addition of fibre reinforcement is also a promising advancement for 3D printing of polymers [71,139,140]. Additive manufacture of polymer fibre composites can transform additive manufacturing into a robust manufacturing process that can produce customised products with significantly improved mechanical properties as compared to using un-reinforced polymers as well as enable 4D printing using fibres to control and manipulate the change of shape or swelling [140]. However, there are still areas that are in need of improvement that include void formation, poor adhesion of fibres and matrix, blockage due to filler inclusion, increased curing time, modelling, and simulation. There are also clear indications that upcoming research related to fibre reinforced additive manufacturing will be driven by the advancement of computers, resources, and analytical tools [71].

## 6. Conclusions

Petrochemical plastics have been used widely since their inception to date. They have remained a preferential choice in food packaging as a result of their properties and low cost of production. However, they have proven to have a grave impact on the environment and its habitants. The food packaging industry is a major consumer of plastic as well as a major source of plastic waste. This has created the need to adopt more sustainable means of food packaging. Using biodegradable polymers will exploit the utilisation of agricultural waste to alleviate the fossil fuel shortage, solve environmental issues involved when using petroleum-based plastics, as well as lead to a circular economy. The interaction between food and the packaging material during processing and storage is important, as are thermal, barrier, and mechanical properties. PLA has emerged as a safe and promising polymer for primary food packaging application. However, it has some inherent drawbacks. To counter its drawbacks, blending with natural fillers and fibres will see the growth in this application domain to produce low cost functional packaging materials which are environmentally friendly. At the end of their useful lifetime, these biodegradable composites can be biologically recycled and serve as a biological nutrient. An increase in the bioplastic applications can also realise employment growth in the sector and have cascading effects that could even lead to the development of rural areas where agricultural waste can be used as a sustainable input resource.

## Figures and Tables

**Table 1:** Some PLA based composites.

1. Filler	2. Composite Fabrication Process	3. Observation	4. Reference
Hemp fibres	Twin extruder, compression and injection moulding	− Increased tensile strength, young’s modulus and impact strength − Fibre treatments with alkali and saline increased tensile and impact strength	[107]
Fine grain filler of native cellulose	Melt-mixing	− Good dispersion of the filler giving an aesthetic appearance, creamy colour and glossy surface − Good thermal stability	[98]
Ceramic food waste from grinding egg shells and mussel shells	Melt-mixing	− High filler amounts of 140 over 100 parts of PLA − Thermoplastic, biodegradable and low carbon footprint composites − Composites do not release volatiles typical of fossil-based plastics that are hormone disruptors or priority air pollutants that pose public health	[66]
Silver skin (waste from roasting coffee beans)	Melt-mixing	− Up to 30%wt% of filler content can be added − Increase in young’s modulus	[103]
Waste from wine production (grape skins, seeds and stalk fragments)	Molding	− Up to 20% of filler can be added − High elastic modulus and impact strength but lower tensile strength − Increase in moisture absorption with increase in filler content	[102]
Cocoa bean shells	Solution casting	− Improved physical properties of the composites − Low levels of food migration and improved barrier properties	[108]

**Table 2:** Comparison of PLA properties to other common polymers used in food packaging. Data from [52,53,111,112,113].

Property/Polymer	LDPE	PET	PLA	PP	PS
Strength (MPa)	10–12	55–79	37–66	15–27	24–60
Elongation at Break (%)	300–500	15–165	0.5–9.2	100–600	1.6–2.5
Oxygen barrier (permeation at 30 °C [*10^−10^ cm^3^(STP)·cm/cm^2^·S·cm Hg])	6.9	0.04	3.3	1.5	2.6
Moisture vapour transmission rate (g-mil/10in.^2^/24 h)	1.0–1.5	2	18–22	0.5	10
Water absorbance (%)	0.005–0.015	0.1–0.2	3.1	0.01–0.1	0.01–0.4
Thermal properties [Glass Transition Temperature-Tg (°C)]	−110	73	55	−20	90
Transparency (Clarity)	High	Excellent	High	Poor	Excellent
Carbon dioxide barrier (permeation)	28	0.2	10.2	5.3	10.5
Chemical resistance	Good	Good	Poor	Good	Good
